# Rodent Lethality Models Are Problematic for Evaluating Antivenoms for Human Envenoming

**DOI:** 10.3389/fphar.2022.830384

**Published:** 2022-02-03

**Authors:** Anjana Silva, Wayne C. Hodgson, Theo Tasoulis, Geoffrey K. Isbister

**Affiliations:** ^1^ Department of Parasitology, Faculty of Medicine and Allied Sciences, Rajarata University of Sri Lanka, Saliyapura, Sri Lanka; ^2^ Monash Venom Group, Faculty of Medicine, Nursing and Health Sciences, Monash University, Clayton, VIC, Australia; ^3^ South Asian Clinical Toxicology Research Collaboration, University of Peradeniya, Peradeniya, Sri Lanka; ^4^ Clinical Toxicology Research Group, University of Newcastle, Newcastle, NSW, Australia

**Keywords:** lethality, snake venom, antivenom, LD50, ED50, rodent, mouse

## Introduction

Snakebite is a major public health problem in the tropics, being closely associated with agricultural economy, poverty and underdevelopment. The neglected nature of the condition over a long period of time has meant that our current understanding of the epidemiology, clinical effects, pathophysiology and the treatment of snakebite has many gaps (Isbister and Silva, 2018). Knowledge of the pathophysiology is essential to understanding the clinical effects of snake envenoming and, more importantly, the response to antivenom.

Snake venoms are a complex mix of different toxins with varying pharmacological properties (Tasoulis and Isbister, 2017). The effects of snake venoms on natural prey species are likely to be different to the effects on non-prey species, such as humans (Richards et al., 2012). Animal models have been important tools for understanding the effects of snake venoms on humans. The action of the whole venom and isolated toxins, and their response to antivenoms, are tested in living laboratory animals, such as rats, mice, and rabbits (*in-vivo*) and tissues isolated from laboratory animals, such as rats, mice, rabbits, frogs, and chickens (*in-vitro*) (Silva and Isbister, 2020). Despite the biochemical and functional complexity of snake venoms observed in animal models, bites by medically important snakes commonly result in a narrow range of acute effects in human envenoming, including local tissue necrosis, venom-induced consumption coagulopathy (VICC), neuromuscular paralysis, acute kidney injury (AKI) and myotoxicity. It is important that animal models used for testing snake toxins, venoms and antivenoms, to better represent human envenoming, mirror the observed clinical effects of human envenoming.

Neutralisation of rodent lethality from snake venoms *in-vivo* has been recommended to manufacturers and regulatory bodies as an essential pre-clinical test of antivenoms by the World Health Organisation (WHO). Mouse lethality prevention assays are considered the “gold standard test” until alternative tests become accepted (World Health Organisation, 2010; Calvete et al., 2018). The WHO further states that the suffering of test animals due to the venom effects until death, or during the 24 or 48 observation period, in the lethality studies outweighed by the larger benefits to humans (World Health Organisation, 2010).

### Why Rodent Lethality Models Are Problematic for Evaluating Antivenoms for Human Envenoming?

The majority of snake venoms that cause paralysis in envenomed humans contain pre-synaptic neurotoxins, which cause paralysis that is, not reversible with antivenom (Silva et al., 2017, 2018). The other major group of snake neurotoxins are the post-synaptically acting α-neurotoxins. These toxins are present in venoms from a range of snakes, including many species that do not result in paralysis in humans (Barber et al., 2013; Youngman et al., 2021). The proportion of α-neurotoxins in snake venoms is known to be associated with the prey selectivity of the venoms (Harris et al., 2020), which further suggests that these particular toxins are animal-specific. The two major types of α-neurotoxins, i.e., long-chain (LαNTx) and short-chain (SαNTx), bind to the same region of the α-subunit of the nAChR, resulting in inhibition of neurotransmission at the skeletal neuromuscular junction. Current knowledge about the relevance of snake venom α-neurotoxins in human envenoming and paralysis is largely based on projections from data generated from rodent, avian, and amphibian pharmacological models (Silva et al., 2017). However, using skeletal muscle from several animals, including rodents, non-human primates and humans, a largely neglected study, dating back to 1985, showed that human nAChR have an exceptionally low affinity for SαNTx compared to LαNTx, while both groups bind with high affinity to mouse nAChRs (Ishikawa et al., 1985). Consistent with these observations, in a functional study using human and rat nAChR, we recently demonstrated that the human nAChR is more resistant to snake SαNTx compared to the rat nAChR, as evidenced by marked differences in the speed of the reversibility of toxin-mediated inhibition of the human nAChR (Silva et al., 2018). The experiments, with and without various mutations of the human and rat nAChR, showed that this species difference is due to the absence of large aromatic amino acid residues at positions 187 and 189 in loop C of the α subunit of the human nAChR. This is in contrast to rats, mice, and nAChRs in other animals commonly used for *in-vitro* testing, including *Torpedo californica*, which possess large aromatic amino acid residues at equivalent positions. However, no such difference was observed between the effects of LαNTx on rat and human nAChRs.

Inhibition of the nAChR only requires the binding of one α-neurotoxin molecule to one of the two ACh binding sites on the receptor. Theoretically, paralysis could develop in humans following a snakebite if the concentration of α-neurotoxins at the neuromuscular junction is such that the nAChRs are inhibited and α-neurotoxin-receptor binding could be sustained, without being rapidly reversed naturally. However, sustaining sufficient α-neurotoxin concentrations at the neuromuscular junction to maintain inhibition of the nAChR depends on the relative abundance of the α-neurotoxins in the particular snake venom as well as the amount of venom injected during the snakebite. This means that snakes injecting large amounts of venom when biting humans, and/or having a higher relative abundance of LαNTx in their venoms, such as some species of cobras (*Naja*) or the king cobra (*Ophiophagus hannah*), can potentially cause post-synaptic neurotoxic paralysis in humans. Snakes containing only SαNTx or only small amounts of LαNTx, will not cause paralysis in humans. SαNTxs are unlikely to be clinically important, except in unique situations such as Philippine cobra (*Naja philippinensis*) envenomings in which there is such a large venom load, human paralysis can still occur, despite a higher relative abundance of SαNTx (∼45%) (Tan et al., 2019). Therefore, assays heavily influenced by the effects of α-neurotoxins in any animal except humans are not representative of paralysis in humans and are problematic for antivenom development. In agreement with this, recent studies have demonstrated that the outcome of rodent lethality assays are heavily influenced by the α-neurotoxin activity of snake venoms, when α-neurotoxins are present in the venoms (Petras et al., 2011; Pruksaphon et al., 2020).

Rodent lethality prevention assays have shown results comparable to human envenoming in some viperine venoms lacking α-neurotoxins, such as carpet vipers (*Echis* sp.) and south American Bushmasters (*Lachecis* sp.) (Gutiérrez et al., 1988; Bard et al., 1994; Diniz-Sousa et al., 2020). Although α-neurotoxins are mostly found in elapids and colubrids, they are present even in some viperid venoms that do not cause neurotoxicity in humans, such as Puff adders (Wang et al., 2020; Youngman et al., 2021) hence the utility of these assays remains narrow.

There are other examples in which animal models do not represent human envenoming. Plasma from several large animals, including rats, were shown to be resistant to concentrations of procoagulant toxins several orders of magnitude greater compared to humans, from viperids such as *Daboia russelii*, *Echis carinatus*, *Callocellasma rhodostoma* and elapids such as *Pseudonaja textillis*, (Maduwage KP. et al., 2016). This means, in order to replicate VICC in rodent models, the animals need to be exposed to very high venom concentrations, which would lead to the death of animals from the primary and secondary effects of venom that are not relevant to humans (i.e., neurotoxicity). In a recent study, acute kidney injury was not able to be replicated in rats with sub-lethal doses of *D. russelii* venom suggesting it to be a poor model for venom-induced acute kidney injury (Wijewickrama et al., 2018). VICC, AKI and thrombocytopaenia only occurred with experimental venom doses that were unrealistically higher than what is observed in actual human envenomings, making it difficult to match the real-life envenoming scenarios in humans (Tan et al., 2012; Romanelli et al., 2021; Thomazini et al., 2021; Yamamoto et al., 2021).

## Discussion

Among the WHO approved list of essential quality control parameters for routine quality control testing of antivenoms, the sole parameter that is used to assess the pharmacological/therapeutic efficacy of antivenom is the lethality neutralisation test (World Health Organisation, 2017; Patra et al., 2021). The WHO guidelines for the production, control and regulation of snake antivenom immunoglobulins, published in 2017, states that “Despite reservations about the physiological relevance of these animal (murine) models to human envenoming and the harm that these *in vivo* assays cause to the animals, they are used by both manufacturers and regulatory authorities worldwide for determining venom lethality (LD_50_) and antivenom neutralizing capacity (ED_50_) as these are currently the only validated means of assessing venom toxicity and antivenom neutralizing potency” (World Health Organisation, 2017). Further, it states “Non-sentient or *in vitro* assays as alternatives to the standard venom LD_50_ and antivenom ED_50_ *in vivo* tests have been promoted. Unfortunately, such systems have not been developed to the point where they can fully replace the above-mentioned preclinical assays.” Further, the WHO report states that “in the absence of effective alternatives, the continued use of experimental animals is still justified by the considerable benefits to human health of these preclinical assays” (World Health Organisation, 2017).

The fundamental assumption behind testing venoms, toxins and antivenoms, using rodent lethality models in relation to human envenoming, is that the “toxins possessing the highest rodent lethality are the most medically important toxins in human envenoming” (Lauridsen et al., 2016; Calvete et al., 2018; Silva and Isbister, 2020). This assumption requires evidence that there is a clear relationship between the outcome of rodent lethality tests and clinical toxicity in human envenoming, in venom doses comparable with bites in human envenomings. However, due to the reasons highlighted above, we argue that the relevance of the death or survival of an animal observed in rodent lethality studies, lethality prevention studies or rescue models, to an envenomed human with snakebite is highly questionable. It is not an appropriate model for characterisation of medically important venoms and pre-clinical testing of snake antivenoms. Rodent lethality-based assays cause enormous suffering to the test animals, so the minimal value of these lethality models does not outweigh the suffering of test animals.

The venom composition of snakes varies enormously resulting in different envenoming syndromes in envenomed humans with different clinical outcomes. Reducing the complexity of the effects of snake venoms in humans to a single parameter, the lethality of an experimental animal, is a gross oversimplification. Efficacy assays need to be based on the ability of the antibodies in antivenoms to bind with the venom toxins *in-vitro* and to neutralise or prevent the clinically relevant effects of snake toxins in human envenoming (Maduwage K. et al., 2016). The efficacy of antivenoms in neutralising the clinically most-important systemic effects of envenoming such as neuromuscular paralysis, VICC and myotoxicity could be successfully tested using *in-vitro* functional assays such as neuromuscular preparations that test the neutralisation of clinically relevant pre-synaptic neurotoxins and post-synaptic toxins by antivenom (Silva et al., 2016b; 2016a) and *in-vitro* procoagulant activity neutralisation assays that test the ability of antivenoms to neutralise the procoagulant effects of venoms on human plasma (Maduwage K. et al., 2016). Although the *in-vitro* procoagulant assays are relatively simple to conduct, the *in-vitro* neurotoxicity assays require organ-bath systems and sufficient technical expertise. More ethically appropriate alternative tests such as embryonated egg models have also been introduced, but need validation across a range of snake venoms as well as the relevance to envenoming syndromes in humans (Sells, 2003; Verity et al., 2021).

Rodent lethality models are relatively simple to conduct hence not resource-intensive. They are easy to interpret and are affordable for developing settings (1). While acknowledging the long history and simplicity of rodent LD_50_ and ED_50_ assays, we emphasise that they measure toxic effects that are not necessarily relevant in humans. They do not sufficiently represent envenoming syndromes in humans and have considerable ethical issues. The lack of alternative models does not justify the use of irrelevant and ethically questionable rodent lethality tests.
